# Neuroprotective Effect of *Uncaria rhynchophylla* in Kainic Acid-Induced Epileptic Seizures by Modulating Hippocampal Mossy Fiber Sprouting, Neuron Survival, Astrocyte Proliferation, and S100B Expression

**DOI:** 10.1155/2012/194790

**Published:** 2011-08-03

**Authors:** Chung-Hsiang Liu, Yi-Wen Lin, Nou-Ying Tang, Hsu-Jan Liu, Ching-Liang Hsieh

**Affiliations:** ^1^Department of Neurology, China Medical University Hospital, Taichung 40402, Taiwan; ^2^Graduate Institute of Acupuncture Science, China Medical University, Taichung 40402, Taiwan; ^3^Acupuncture Research Center, China Medical University, Taichung 40402, Taiwan; ^4^School of Chinese Medicine, China Medical University, Taichung 40402, Taiwan; ^5^Department of Chinese Medicine, China Medical University Hospital, Taichung 40402, Taiwan

## Abstract

*Uncaria rhynchophylla* (UR), which is a traditional Chinese medicine, has anticonvulsive effect in our previous studies, and the cellular mechanisms behind this are still little known. Because of this, we wanted to determine the importance of the role of UR on kainic acid- (KA-) induced epilepsy. Oral UR for 6 weeks can successfully attenuate the onset of epileptic seizure in animal tests. Hippocampal mossy fiber sprouting dramatically decreased, while neuronal survival increased with UR treatment in hippocampal CA1 and CA3 areas. Furthermore, oral UR for 6 weeks significantly attenuated the overexpression of astrocyte proliferation and S100B proteins but not *γ*-aminobutyric acid A (GABA_A_) receptors. These results indicate that oral UR for 6 weeks can successfully attenuate mossy fiber sprouting, astrocyte proliferation, and S100B protein overexpression and increase neuronal survival in KA-induced epileptic rat hippocampus

## 1. Introduction

Epilepsy is characterized as a condition of brain imbalance, especially in the hippocampus, with unpredictable discharge and seizures. In clinical studies, 30% of patients with uncontrolled seizures and using current antiepileptic drugs do not have curative therapy. Many antiepileptic drugs act as antiexcitatory or inhibitory agents to suppress seizure occurrence. These results in side effects on cognition and memory [1]. Developing more specific antiepileptic drugs that target cellular mechanisms while maintaining normal brain function is crucial. Glutamate is one of the excitatory neurotransmitters which bind to N-methyl-D-asparate (NMDA), alpha amino-3-hydroxy-5-mehtyl-4-isoxazole (AMPA), and kainic acid (KA) receptors to enhance neuronal activity through sodium influx. Activation of KA subtype of glutamate receptors by glutamate or KA have been reported to contribute to the epilepsy process. Recent scientific studies have indicated that intraperitoneal (i.p.) injection of KA can successfully induce epilepsy in both rats and mice with accompanying phenomena similar to that in human temporal lobe epilepsy [2]. 

Glutamate is one of the common excitatory neurotransmitters in the mammalian central nervous system (CNS). It is released from presynaptic terminals and binds to glutamate receptors [3]. Glutamate receptor activation causes a large influx of sodium ions which excite neurons. One of the major inhibitory neurotransmitters in the CNS is *γ*-aminobutyric acid (GABA). The GABA_A_ receptor is one of the most important inhibitory neurotransmitters in the brain and is a focus of study in animal models of epilepsy [4, 5]. Activation of GABA_A_ receptors is used in the development of antiepileptic drugs, including benzodiazepines, gabapentin, and barbiturates [6, 7]. Imbalanced neuronal network damage in the mammalian brain is highly associated with neurological and neurodegenerative diseases such as epilepsy [8], Parkinson's [9], and Huntington's disease [10]. Epilepsy often occurs in patients suffering from recurrent seizures and it is usually associated with an imbalance of excitatory and inhibitory neurons in the CNS [11]. 

Epilepsy is usually associated with neuronal loss [12], astrocyte proliferation [13], mossy fiber sprouting [14], and synaptic reorganization in the hippocampus. The neuron loss is mainly observed in the CA3, CA1, and hilus areas in the hippocampus [15]. These alterations, especially mossy fiber sprouting, are highly associated with spontaneous recurrent seizures in humans and in epileptic animal models. The most frequent pathologic techniques to investigate axon sprouting in epileptic animal models using Timm's stain [16]. Axon sprouting from dentate granular cells increases neuronal excitability by forming numerous novel synapses leading to seizure generation [17]. Recent studies have reported that regular exercise increases the synapses in the hippocampal dentate gyrus (DG) to increase memory formation [18]. Mice with pilocarpine-induced epilepsy had significant mossy fiber sprouting in the inner molecular layer of the DG. Animals with pilocarpine-induced epilepsy also developed spontaneous seizures at 3 weeks after induction. However, whether or not these phenomena are directly involved in the epileptogenic process or are just an indirect secondary consequence of excitotoxicity from recurrent seizures is unclear [19].

Upregulation of glial fibrillary acidic protein (GFAP) and S100B protein was observed in the late phase of epileptic animal models but not during recurrent seizure onset [20]. Recently, glial cells and astrocytes have been implicated in pain sensation, immune response, and neural information processing. Astrocytes have also been reported as functional receptors as neurons and can regulate astrocyte function [21]. Elevation of the intracellular Ca^2+^ concentration in cultured astrocytes can induce glial cells to release glutamate [22]. Glial cells and astrocytes can release neurotransmitters to modulate neuron excitability and plasticity [23]. S100 proteins, first isolated from the brain in 1965, are low-molecular weight proteins that have calcium-binding properties [24]. S100B proteins are highly expressed in the CNS to enhance neurite outgrowth [25] and stimulate astrocyte proliferation* in vitro* [26]. Age-related increases in astrocytes have been observed in the hippocampus as determined with the astrocyte specific marker, GFAP. S100B can also increase intracellular free calcium concentrations to regulate neuron excitability [27]. Chronic epilepsy causes overexpression of S100B by astrocytes from neuronal damage or dysfunction [28]. 

UR, a Chinese medicinal herb, is used to decrease hyperfunction of the liver, dizziness, and epilepsy. UR also has an antiepilepsy effect in KA-induced seizures in rats [29]. The alkaloid fragments of UR, including *rhynchophylline*, *isorhynchophylline*, and *isocorynoxeine*, have been identified as having a protective function that prevent neurons from glutamate-induced cell death [30]. UR also protected neurons from apoptosis and participated in neuronal protection [31] by inhibiting c-Jun kinase phosphorylation and nuclear factor-*κ*B (NF-*κ*B) activity in KA-induced epileptic rats [32]. 

To investigate the roles and mechanisms of oral UR on KA-induced long-term seizures, we investigated whether UR could ameliorate KA-induced epileptic seizures. The alkaloid fragment of UR is known for its antiepileptic effect in clinical therapy through its ability to inhibit abnormal recurrent discharges and further induce apoptosis. We used animal behaviors, immunohistochemistry, and Western blotting techniques to verify the regulatory role of UR in KA-induced epilepsy. We next tested the expressions of NeuN, GFAP, S100B, and GABA_A_ in control, KA-induced, and UR-treated groups. Overall, oral UR for 6 weeks significantly attenuated epileptic seizure and simultaneously increased neuron survival. It also attenuated GFAP and S100B protein levels but not GABA_A_ receptors.

## 2. Materials and Methods

### 2.1. Animals

Male Sprague-Dawley (SD) rats weighing 200–300 g were used in this study. Rats fasted overnight with free access to water. Usage of the animals was approved by the Institute Animal Care and Use Committee of China Medical University and followed the Guide for the Use of Laboratory Animals (National Academy Press).

### 2.2. Extraction of UR

The UR (Rubiaceae, UR jacks) in the present study was purchased from China and authenticated by Chiu-Lin Tsai (director, division of Traditional Medicine Pharmacy, China Medical University Hospital, Taiwan). The UR was extracted by the Koda Pharmaceutical Company (Taoyuan, Taiwan). The voucher specimen was kept in the neuroscience laboratory room of China Medical University. Eight kg of crude UR was extracted with 64 kg of 70% alcohol by boiling for 35 min. These extracts were filtered, freeze-dried, and then stored in a drier box. The total yield was 566.63 g (7.08%). The freeze-dried extracts of UR were qualified by a high performance liquid chromatography (HPLC) system (interface D-700, Pump L-7100, UV-Vis Detector L-7420; Hitachi Instruments Service Co. Ltd., Ibaraki-ken, Japan) using *rhynchophylline* (Matsuura Yakugyo Co. Ltd., Japan) as a standard from the Koda Pharmaceutical Company. Each gram of freeze-dried extract contained 1.81 mg of pure alkaloid component of UR. The dose response for this compound was reported in our previous study [29]; hence, we used this effective dose for all experiments in this study.

### 2.3. Establishment of Epileptic Seizure Model

These experiments used 36 SD rats. Four days prior to the electroencephalogram (EEG) and electromyogram (EMG) recordings, all rats underwent stereotactic surgery with chloral hydrate (400 mg/kg, i.p.) anesthesia. The scalp was then incised from the midline and the skull was exposed. Stainless steel screw electrodes were implanted on the dura over the bilateral sensorimotor cortices to serve as recording electrodes. A reference electrode was placed in the frontal sinus. Bipolar electrical wires were placed on the neck muscles for EMG recordings. Electrodes were connected to an EEG and EMG-monitoring machine (MPlOOWSW, BIOPAC System, Inc., Calif, USA). The epileptic seizures were confirmed by behavior observation (including wet dog shakes, paw tremors, and facial myoclonia under a freely moving and conscious state), and epileptiform discharges on EEG recordings. 

The study was divided into experiment 1 and experiment 2. Each experiment included 18 rats. Each experiment was divided into three groups of 6 rats as follows: (1) the control group with phosphate buffered saline (PBS) i.p. only without KA, (2) the KA group with KA at 12 mg/kg i.p. only, and (3) the UR group receiving oral UR at 1 g/kg, 5 days/week continuously for 6 weeks starting the next day after KA injection. All the rats were sacrificed at 6 weeks after KA injection and the brains were removed for Timm's stain and for GFAP and NeuN immunohistochemistry staining (IHC) studies in experiment 1; and for S100B and GABA_A_ IHC studies in experiment 2.

### 2.4. Timm's Stain

The rats were perfused with 0.37% sodium sulfide solution (1.17 g of Na_2_S·9H_2_O, 1.19 g of NaH_2_PO_4_·H_2_O per 100 mL) and 4% paraformaldehyde transcardially under anesthesia with chloral hydrate (400 mg/kg) i.p. The brain tissues were postfixed in 4% paraformaldehyde. Two days prior to sectioning, the brain tissues were immersed in a 30% sucrose solution diluted in phosphate buffer solution (PBS). After immersion, the brains were removed from the sucrose solution and were embedded in the tissue freezing medium at −80°C and stored at −20°C. The brains were cryostat-sectioned (40-*μ*m coronal sections for Timm's staining and 16-*μ*m coronal sections for immunohistochemical staining). The 40- and 16-*μ*m sections were alternately processed. The 40-*μ*m sections were immersed in the dark in developing solution. This solution consisted of 5.1 g of citric acid, 4.7 g of sodium citrate, and 3.4 g of hydroquinone dissolved in 80 mL of water and then added to 180 mL of 50% Arabic gum. Then, 1 mL of 17% silver nitrate solution was added to this mixture just before the start of the developing process. The sections were checked occasionally until proper staining was evident, then washed in running water, dehydrated with a graded series of ethanol solutions for 15 min (75% for 5 min, 95% for 5 min, and finally, 99% for 5 min) and xylene for 5 min, and coverslipped. 

To quantify mossy fiber sprouting in the supragranular molecular layer of the dentate gyrus, the staining intensity was measured on a picture of Timm's stained sections. Briefly, stained coronal sections taken 3.64–4.10 mm from the bregma were viewed under a Zeiss microscope, and pictures were captured by a Nikon digital camera. Utilizing image analysis software (Image-Pro Plus, Media Cybernetics, Inc., Silver Spring, Md, USA), the pictures were converted to 8-bit gray scale images. Optical density (OD) measurements based on an average gray-scale value (0–255 pixels unit) were obtained for two parallel lines along the inner and outer edges of the inner molecular layer (IML). A lower value of OD corresponded to a darker image. The inner edge of the IML is the place where aberrant mossy fibers terminate, while the outer edge of the IML was used to determine a background value. The difference in OD between the inner and outer edge of the IML of each section was defined by

(1) $$\frac{\left({\text{OD}}_{o}-{\text{OD}}_{i}\right)}{\left[{\text{OD}}_{o}+{\text{OD}}_{i}/2\right]},$$

where OD_*o*_ is the OD value of the outer edge of the IML (background value) and OD_*i*_ is the OD value of the inner edge of the IML (sprouting mossy fiber terminals). The difference in OD, therefore, represents the extent of mossy fiber sprouting in the IML. The greater the difference in the OD, the higher is the density of sprouting mossy fibers.

### 2.5. Immunohistochemistry staining

Sections were first washed twice (5 min each) in 0.1 M TRIS buffer (pH 7.6) and treated with 1% H_2_O_2_ made in 0.1 M TRIS buffer for 30 min to inhibit the endogenous peroxidase activity. Then, they were washed in 0.1 M TRIS buffer for 5 min; TRIS A, for 10 min (TRIS A: 0.1% Triton X-100 dissolved in 0.1 M TRIS buffer), and TRIS B, for 10 min (TRIS B: 0.1% Triton X-100 and 0.005% bovine serum albumin (BSA) in 0.1 M TRIS buffer). Then, the sections were blocked in normal goat serum (45 min), washed in TRIS A and then TRIS B (10 min each), and then incubated with antibody against NeuN (1 : 1000, Chemicon, USA), GFAP (1 : 200, CALBIOCHEM, Germany), GABA_A_ (1 : 1000, Millipore, USA), and S100B (1 : 1000, Novus Biologicals, USA) overnight at 4°C. On the following day, sections were washed with TRIS A (10 min), then, TRIS B (10 min), incubated with biotinylated goat antirabbit immunoglobulin (Ig) G (45 min), washed with TRIS A (10 min), washed with TRIS D (0.1% Triton X-100 and 0.005% BSA in 0.5 M TRIS buffer; 10 min), incubated with avidin-biotin horseradish peroxidase complex (1 hour), washed three times with 0.1 M TRIS buffer for 5 min each, developed in 3,3-diaminobenzidine (DAB, 1-2 min), washed three times with 0.1 M TRIS buffer (5 min each), and finally, incubated with 0.1 M TRIS buffer to stop the reaction. All sections were counterstained with hematoxylin solution, washed three times with 0.1 M TRIS buffer, dried, and coverslipped.

### 2.6. Statistical Analysis

The data are presented as mean ± SD, and a one-way ANOVA with Scheffe's *post hoc* test was used to examine differences between the groups. *P* < 0.05 was considered as statistically significant.

## 3. Results

### 3.1. Induction of Epileptic Seizures through Intraperitoneal Injection of KA as Assessed by EEG

**Figure 1:**
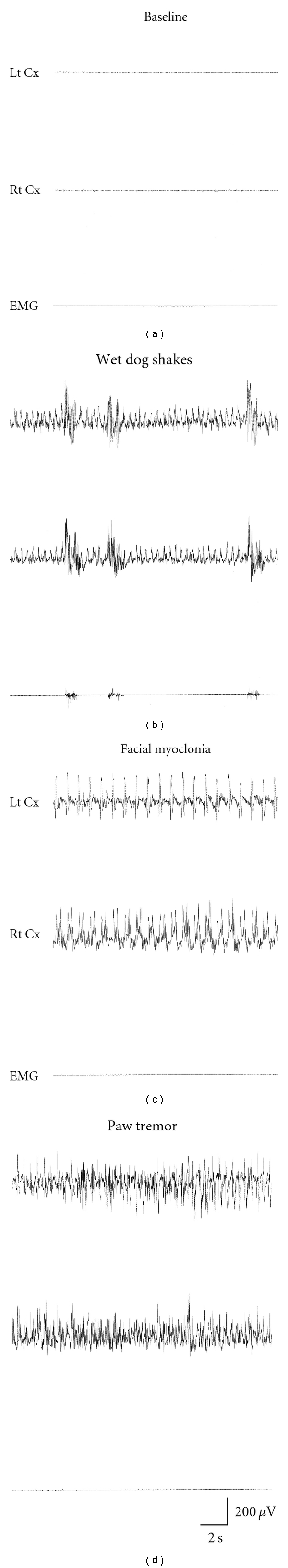
The alteration of electroencephalographic (EEG) signals in KA-injected animals. Basal EEG activity from the sensorimotor cortex was characterized by 6–8 Hz activity in rats when awake (a). KA-induced temporal lobe seizures, including wet dog shakes (WDS) with intermittent polyspike-like activity (b), facial myoclonia with continuous sharp waves (c) and paw tremor (PT) with continuous spike activity (d). Lt Cx = EEG recording from the left sensorimotor cortex; Rt Cx = EEG recording from the right sensorimotor cortex; EMG = EMG recording from the neck muscle.

Epilepsy is one of the major CNS diseases with imbalanced nerve discharge resulting in overexcitability in the cerebral cortex. An epileptic animal model was used to test the effect of oral UR. Epileptic seizures were induced in both the left and right cortex in 24 SD rats using a KA injection (12 mg/kg, i.p.). Three major types of seizures were induced with their own characteristic EEG activity. Limbic motor signs, such as wet dog shakes, paw tremor, and facial myoclonia, were recorded in all epileptic rats.  Figure 1(a) shows baseline recordings. Wet dog shakes-induced electrical waveform was shown by intermittent polyspike-like EEG activity (Figure 1(b)). Facial myoclonia-induced electrical waveform was indicated by characteristic continuous sharp EEG activity (Figure 1(c)). Paw tremor-induced electrical waveform was defined as continuous spike EEG activity (Figure 1(d)). Electromyogram (EMG) responses were also recorded.

### 3.2. Long-Term Oral UR Reduced Mossy Fiber Sprouting in KA-Induced Epileptic Rat

**Figure 2:**
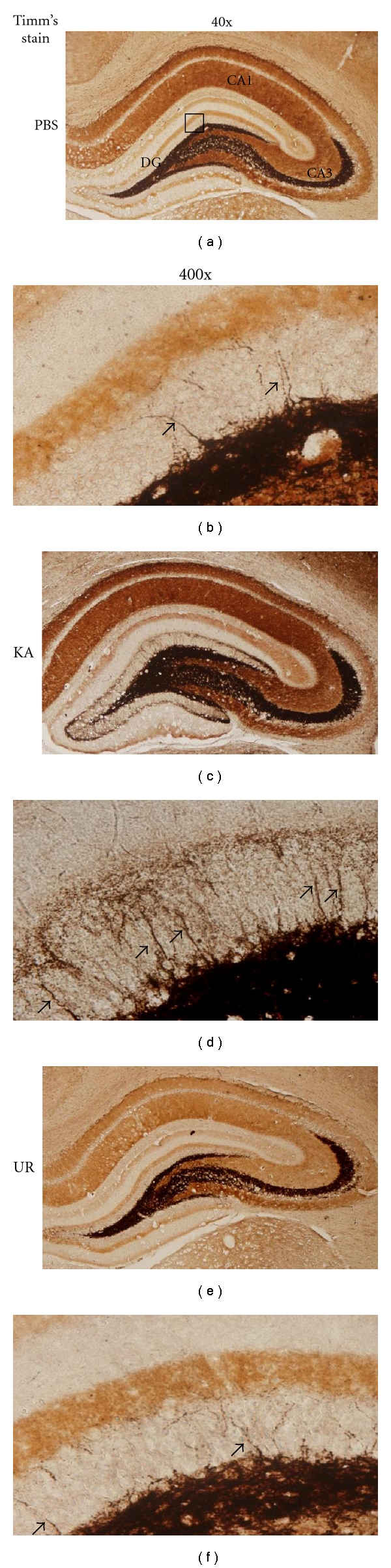
Timm's stain in hippocampal slices from PBS, KA, and UR pretreated groups. (a) whole hippocampus in PBS group (b) DG molecular layer in PBS group (c) whole hippocampus in KA-induced group. (d) DG molecular layer in KA-induced group (e) whole hippocampus in UR-treated group (f) DG molecular layer in UR-treated group. Timm's staining indicated with arrows (brown). The left panel was captured at 40x magnification while the right panel was at 400x magnification.

Mossy fiber sprouting accounts for recurrent neural discharge in the hippocampus. Timm's staining was used to determine whether oral UR could reverse this phenomenon in epileptic rats. The results indicated that little sprouting was present in the DG molecular layer in the PBS-injected group (Figures 2(a) and 2(b), 10.0 ± 0.8%). However, sprouting greatly increased in rats having a KA injection (Figures 2(c) and 2(d), 49.8 ± 2.7%, *n* = 6). This sprouting phenomenon was reduced in rats with long-term UR administration (Figures 2(e) and 2(f), 17.3 ± 1.1%, *n* = 6). KA-induced epileptic rats demonstrated mossy fiber sprouting and recurrent seizures. Long-term oral UR use successfully reduced this sprouting at 6 weeks after KA-injection in the hippocampal DG molecular layer.

### 3.3. UR Improves Neuron Survival in Rat Hippocampus in KA-Induced Epilepsy

**Figure 3:**
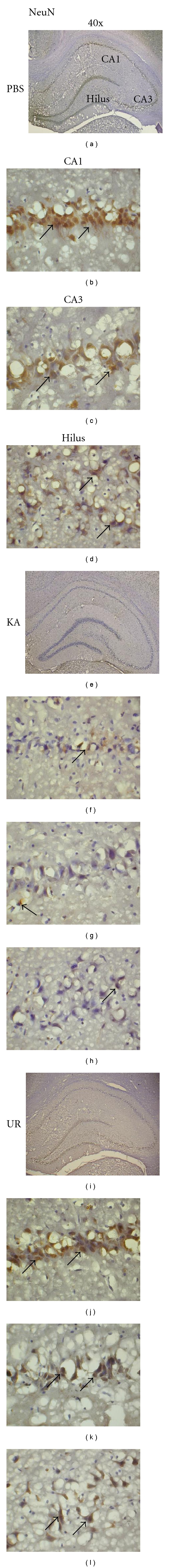
Immunohistochemistry staining of Hematoxylin and Eosin (HE) and NeuN in hippocampal slices from PBS, KA-induced, and UR-pretreated groups. HE (blue) and NeuN (brown) positive staining in whole hippocampus (a), CA1 (b), CA3 (c), and Hilus regions in PBS group. HE (blue) and NeuN (brown) positive staining in whole hippocampus (e), CA1 (f), CA3 (g), and Hilus (h) regions in KA-induced group. HE (blue) and NeuN (brown) immunostaining in whole hippocampus (i), CA1 (j), CA3 (k), and Hilus (l) regions in UR-treated group. The left panel was captured at 40x magnification, while the others were at 400x magnification.

To characterize the curative effect of UR in epileptic rats after KA injection, the neuronal marker NeuN was employed. No significant hippocampal neuron loss occurred after PBS injection (Figure 3(a)) in either the CA1 (Figure 3(b), 242.1 ± 30.3% neurons/field), CA3 (Figure 3(c), 89.0 ± 7.6% neurons/field), or Hilus regions (Figure 3(d), 98.7 ± 7.8% neurons/field). After KA injection, the hippocampal neurons were decreased using NeuN immunohistochemistry staining (Figure 3(e)). Importantly, the neuron loss phenomena were mainly observed in the CA1, CA3, and the Hilus areas (Figures 3(f)–3(h), 34.2 ± 3.1%, 30.2 ± 6.1%, 35.3 ± 7.4% neurons/field, *n* = 6, resp.). Long-term oral UR use for 6 weeks decreased hippocampal neuron death from i.p. KA injection (Figure 3(i)), which mainly occurred in the CA1, CA3, and Hilus areas (Figures 3(j)–3(l), 113.5 ± 16.4%, 87.3 ± 13.4%, and 77.7 ± 9.3% neurons/field, compared with control group, *n* = 6, resp.). These results suggest that KA i.p. might increase neuron death in the rat hippocampus which can be reversed by long term UR administration.

### 3.4. UR Attenuates Hippocampal Astrocyte Proliferation in KA-Induced Epilepsy

**Figure 4:**
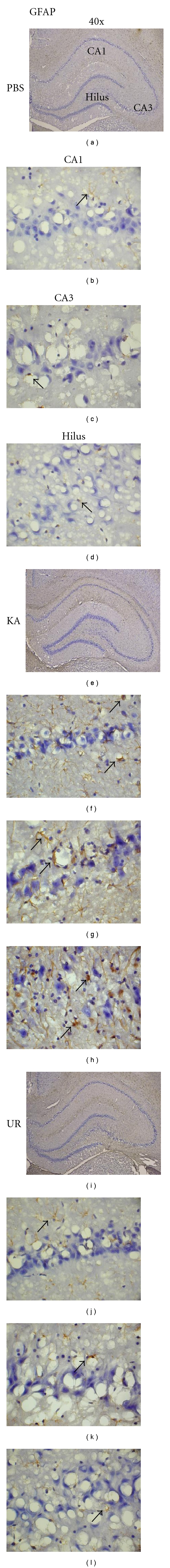
Immunohistochemistry staining of HE and GFAP in hippocampal slices from PBS, KA-induced, and UR-treated groups. HE (blue) and GFAP (brown) positive staining in whole hippocampus (a), CA1 (b), CA3 (c), and Hilus (d) regions in PBS group. HE (blue) and GFAP (brown) positive staining in whole hippocampus (e), CA1 (f), CA3 (g), and Hilus (h) regions in KA-Induced group. HE (blue) and GFAP (brown) positive staining in whole hippocampus (i), CA1 (j), CA3 (k), and Hilus (l) regions in UR-treated group. The left panel was captured at 40x magnification, while the others were at 400x magnification.

Next, the roles of astrocytes in the hippocampus from KA-induced epileptic rats were tested. Astrocyte proliferation was normal in the PBS-injected group (Figure 4(a)). They were distributed in the CA1, CA3, and Hilus areas (Figures 4(b)–4(d), 20.7 ± 3.2%, 15.8 ± 3.2%, and 53.5 ± 4.3% astrocyte/field). Astrocytes increased in the hippocampal neurons of KA-induced rats (Figure 4(e)), especially in the CA1, CA3, and Hilus areas (Figures 4(f)–4(h), 112.0 ± 11.9%, 56.8 ± 7.5%, and 146.5 ± 7.4% astrocyte/field, *P* < 0.01, *n* = 6, resp.). This phenomenon suggested that KA-induced epilepsy was accompanied by astrocyte proliferation. When UR was used to reduce KA-induced symptoms, the results indicated that the administration of oral UR for 6 weeks attenuated astrocyte proliferation in the hippocampus (Figure 4(i)), especially in the CA1, CA3, and Hilus areas (Figures 4(j)–4(l)), 58.0 ± 7.1%, 28.3 ± 4.6%, and 72.3 ± 5.1% astrocyte/field, *n* = 6, resp.) Serial results implied that astrocyte proliferation increased in the KA-induced group and that oral application of UR can reverse this phenomenon.

### 3.5. UR Decreases Hippocampal S100B Protein Overexpression in KA-Induced Epilepsy

**Figure 5:**
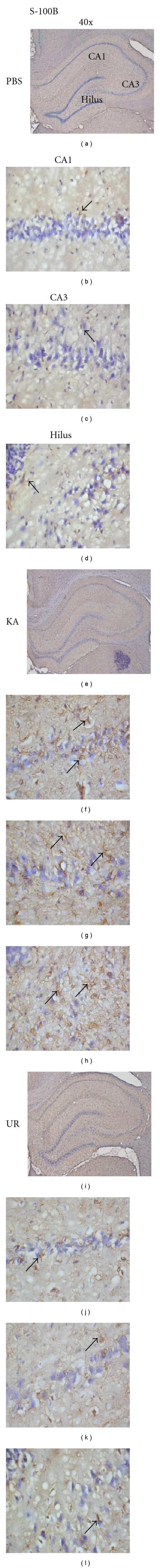
Immunohistochemistry staining of HE and S100B in hippocampal slices from PBS, KA-induced, and UR-treated groups. HE (blue) and S100B (brown) positive staining in whole hippocampus (a), CA1 (b), CA3 (c), and Hilus (d) regions in PBS group. HE (blue) and S100B (brown) positive staining in whole hippocampus (e), CA1 (f), CA3 (g), and Hilus (h) regions in KA-Induced group. HE (blue) and S100B (brown) positive staining in whole hippocampus (i), CA1 (j), CA3 (k), and Hilus (l) regions in UR-treated group. The left panel was captured at 40x magnification, while the others were at 400x magnification.

Several studies have shown that S100B proteins are involved in the development of epilepsy, but the relationship between UR and S100B is unclear. Thus, the roles of S100B proteins in KA-induced epileptic rats were assessed. The expression of S100B proteins were commonly distributed in the rat hippocampus (Figure 5(a)). The S100B proteins were observed in the CA1, CA3, and Hilus areas (Figures 5(b)–5(d), 33.2 ± 6.6%, 19.2 ± 5.5%, and 50.2 ± 7.9%, *n* = 6). The results indicated that KA injection can induce the overexpression of S100B proteins (Figure 5(e)) in the CA1, CA3, and Hilus areas (Figures 5(f)–5(h), 121.2 ± 11.8%, 58.8 ± 5.4%, 119.5 ± 11.8%, *P* < 0.01, *n* = 6, resp.). Further oral administration of UR significantly reduced this phenomenon (Figure 5(i)). The reverse pattern was observed in the CA1, CA3, and Hilus areas (Figures 5(j)–5(l)), 65.3 ± 10.3%, 28.2 ± 3.2%, and 61.5 ± 7.2%, *n* = 6, resp.).

### 3.6. Neuroprotective Role of Oral UR is GABA_A_ Receptor Independent

**Figure 6:**
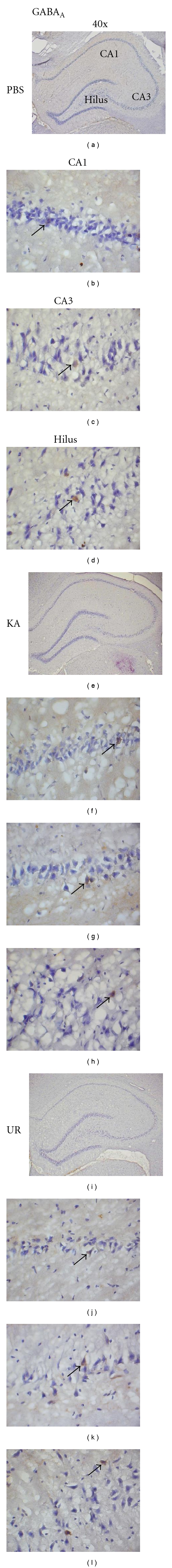
Immunohistochemistry staining of HE and GABA_A_ in hippocampal slices from PBS, KA-induced, and UR-treated groups. HE (blue) and S100B (brown) positive staining in whole hippocampus (a), CA1 (b), CA3 (c), and Hilus (d) regions in PBS group. HE (blue) and GABA_A_ (brown) positive staining in whole hippocampus (e), CA1 (f), CA3 (g), and Hilus (h) regions in KA-Induced group. HE (blue) and GABA_A_ (brown) positive staining in whole hippocampus (i), CA1 (j), CA3 (k), and Hilus (l) regions in UR-treated group. The left panel was captured at 40x magnification, while the others were at 400x magnification.

Next, the expression of GABA_A_ receptors was investigated, since they have been associated with epilepsy. Our results showed that GABA_A_ receptors were present in the rat hippocampus following PBS-injection (Figure 6(a)). High magnification pictures showed that GABA_A_ were distributed in the CA1, CA3, and Hilus areas (Figures 6(b)–6(d), 10.7 ± 2.2%, 5.8 ± 0.5%, and 4.7 ± 0.8%, *n* = 6). The GABA_A_ receptor levels did not change during KA-induced epilepsy in the hippocampus, even in the CA1, CA3, and Hilus areas (Figures 6(e)–6(h), 9.5 ± 3.3%, 4.8 ± 0.9%, and 3.5 ± 0.7%, *P* > 0.05, *n* = 6, resp.). The GABA_A_ receptors were also unchanged after oral administration of UR for 6 weeks (Figure 6(i)). Higher magnifications of the CA1, CA3, and Hilus areas are also presented (Figures 6(j)–6(l), 9.8 ± 2.2%, 6.7 ± 2.0%, and 7.2 ± 2.2%, *n* = 6, resp.). These results suggest that long-term oral UR administration does not alter the expression of GABA_A_ receptors in the hippocampus in KA and UR treated rats.

## 4. Discussion

Here, we used an epileptic animal model to investigate the effect of UR on KA-induced epileptic rats. Three major types of epileptic seizures were recorded and presented, such as wet dog shakes, paw tremors, and facial myoclonia. We used this model to identify the curative role of UR after 6 weeks of oral administration. Our results showed that mossy fiber sprouting increased in the hippocampus of rats receiving KA injections. The phenomenon was reduced with oral UR administration. Oral UR increased the hippocampal neuron survival as shown by NeuN immunostaining. Oral UR also decreased astrocyte proliferation and S100B protein expression. Furthermore, oral UR did not alter the expression of GABA_A_ receptors. Taken together, these results suggest that oral administration of UR has neuroprotective effect in KA-induced epileptic seizures accompanied with increased hippocampal neuron survival. Oral administration of UR can also attenuate mossy fiber sprouting, astrocyte proliferation, and S100B protein expression but not GABA_A_ receptor expression.

Hippocampal mossy fibers, which are the axons from DG granule cells, innervate into dendrites of CA3 principal cells and even inhibitory interneurons. The mossy fibers sprouting are crucial in epilepsy process in hippocampus [1]. Mossy fiber sprouting from recurrent synapses with other granule cells is necessary to increase the excitability leading to epileptic seizures. The classical pathology for both clinical temporal lobe seizures and epileptic animal models is mossy fiber sprouting in the hippocampus as demonstrated with Timm's staining [14]. The mossy fiber sprouting was always associated with granule cell hyperexcitability, epilepsy, learning, and cognitive impairment [14]. Significant neuronal loss was reported in the hippocampus of epileptic animals, including the CA3 and the hilus of the DG [33]. Our results also suggest that oral UR administration can reduce KA-induced epileptic seizures through attenuating mossy fiber sprouting. These results suggest that oral UR administration is a potential candidate for the clinical therapy of epilepsy with mechanisms that operate by attenuating mossy fiber sprouting.

More and more studies have identified astrocytes as active partners in neural information processing. Novel techniques, such as advanced electrophysiological and Ca^2+^ imaging, have revealed that astrocytes have functional ion channels and transmitter receptors like neurons [13]. In epileptic animal models with long-term epilepsy, GFAP immunoreactivity increased with the seizure process [20]. S100B proteins are low-molecular weight proteins which often increase in chronic epilepsy [28], Alzheimer's disease [34], and head trauma [35]. S100B is highly expressed in astrocytes in the CNS, especially in the hippocampus [36]. S100B acts on a variety of CNS neurons to increase calcium concentration and turn on the active phospholipase C and IP3. The overexpression of S100B protein during the epileptic process suggests a principle role for S100B [37]. This implies that downregulating the elevated astrocytes and S100B has a potential in epileptic therapy. Here, we reported that astrocytes and S100B proteins significantly increased during KA-induced epilepsy.

Epileptic seizures, based on excitatory and inhibitory imbalance, can be induced by several methods. Overactivation of excitatory neurotransmitter receptors, such as by using KA injection, has been used to activate kainic receptors which can lead to epileptic seizures [29]. Others have reported that blocking inhibitory GABA_A_ receptors, such as by using pilocarpine, can also be used to successfully induce seizures [38]. Several GABA_A_ receptor agonists, such as carbamazepine and gabapentin, have been used to treat epileptic seizures. In animal models, the alteration of GABA_A_ receptors involved in epileptic seizures is controversial. Synaptically localized GABA_A_ receptors were unchanged, as indicated by the immunoreactivity of postsynaptic GABA_A_ receptors [39]. Vivash et al. reported that GABA_A_ receptor density was reduced in epilepsy rats [40]. Here, we suggest that the use of oral UR for 6 weeks can dramatically reduce epileptic symptoms without changing GABA_A_ receptor density. Thus, we cannot rule out the participation of GABA_A_ receptors in KA-induced seizures, even if no alteration in receptor density occurred. This result agrees with other studies claiming that the GABA_A_ receptor-mediated currents were changed and not the receptor density [41]. Accordingly, the regulation of tonic inhibition plays a more important role in epilepsy than a general alteration of GABA_A_ receptors.

UR, a Chinese herb, has been used for anticonvulsive effects and the treatment of epileptic seizures. The alkaloid component of UR is composed of *rhynchophylline*, *isorhynchophylline*, *corynoxeine*, *hirsutine*, and *hirsuteine*. UR can also protect hippocampal neurons from cell death. The cell death and apoptosis-related genes such as c-jun, bax, and p53 were attenuated when hippocampal neurons were pretreated with the alkaloid component of UR [32]. Our previous study demonstrated that the alkaloid component of UR can ameliorate KA-induced lipid peroxide in vitro and in animal behavior, such as wet dog shakes, paw tremors, and facial myoclonia [29].

## 5. Conclusions

In summary, we suggest that oral UR administration for 6 weeks can significantly reduced mossy fiber sprouting, an indicator for recurrent epilepsy. We further suggest that oral use of UR can protect hippocampal neurons from cell death by using NeuN immunostaining. Oral UR can also prevent hippocampal neuronal death with reduced GFAP and S100B proteins but GABA_A_ receptors.
